# Function and Regulation of the C4-Dicarboxylate Transporters in *Campylobacter jejuni*

**DOI:** 10.3389/fmicb.2017.00174

**Published:** 2017-02-07

**Authors:** Marc M. S. M. Wösten, Chris H. A. van de Lest, Linda van Dijk, Jos P. M. van Putten

**Affiliations:** ^1^Department of Infectious Diseases and Immunology, Utrecht UniversityUtrecht, Netherlands; ^2^Department of Biochemistry and Cell Biology, Utrecht UniversityUtrecht, Netherlands

**Keywords:** *Campylobacter jejuni*, C4-dicarboxylates transporters, DctA, Dcu, gene regulation, metabolism, RacRS

## Abstract

C4-dicarboxylates are important molecules for the human pathogen *C.jejuni*, as they are used as carbon and electron acceptor molecules, as sugars cannot be utilized by this microaerophilic organism. Based on the genome analysis, *C. jejuni* may possess five different C4–dicarboxylate transporters: DctA, DcuA, DcuB, and two homologs of DcuC. Here, we investigated the regulation and function of various C4–dicarboxylate transporters in *C. jejuni*. Transcription of the *dctA* and *dcuC* homologs is constitutive, while *dcuA* and *dcuB* are both directly regulated by the two-component RacR/RacS system in response to limited oxygen availability and the presence of nitrate. The DctA transporter is the only C4-dicarboxylate transporter to allow *C. jejuni* to grow on C4-carbon sources such as aspartate, fumarate, and succinate at high oxygen levels (10% O_2_) and is indispensable for the uptake of succinate from the medium under these conditions. Both DcuA and DcuB can sequester aspartate from the medium under low-oxygen conditions (0.3% O_2_). However, under these conditions, DcuB is the only transporter to secrete succinate to the environment. Under low-oxygen conditions, nitrate prevents the secretion of succinate to the environment and was able to overrule the phenotype of the C4-transporter mutants, indicating that the activity of the aspartate–fumarate–succinate pathway in *C. jejuni* is strongly reduced by the addition of nitrate in the medium.

## Introduction

Bacteria utilize C4-dicarboxylates such as fumarate, succinate, malate, and aspartate when sugars or related compounds are not available (Janausch et al., 2002). C4-dicarboxylates serve as carbon and energy source and are oxidized to CO_2_ in the citric acid cycle under aerobic conditions. Under anaerobic conditions fumarate, malate, and aspartate are taken up into the cell. Malate and aspartate are reduced to fumarate, which is used as electron acceptor in the fumarate respiration pathway where it is converted to succinate. Succinate cannot be further metabolized by most bacteria due to the lack of a functional citric acid cycle under these conditions and is excreted.

The microaerophilic Gram-negative bacterium *Campylobacter jejuni* is the most common cause of food-borne bacterial gastroenteritis worldwide. Despite the medical and public health importance of *Campylobacter* infection, it is remarkable that *C. jejuni* is one of the least understood enteropathogens. *C. jejuni* possesses a highly branched electron transport chain, which allows both aerobic and anaerobic respiration (Kelly, 2008). Most *C. jejuni* strains cannot utilize sugars (Parkhill et al., 2000; Pearson et al., 2007; Stahl et al., 2011) and it seems that selected amino acids and C4-dicarboxylates act as primary energy source (Guccione et al., 2008; Zientz et al., 1999). It remains largely unknown how the transport and regulation of C4-dicarboxylates occurs in *C. jejuni*.

Five C4-dicarboxylate carriers, DctA, DcuAB, DcuC, CitT, and DctPQM, are known to transport C4-dicarboxylates from the periplasm across the inner membrane into bacteria (Janausch et al., 2002). In *Escherichia coli* DctA is a C4-dicarboxylate/H+ or Na+ cation symporter that catalyses the uptake of C4-dicarboxylates during aerobic growth. During anaerobiosis the transcription of the *dctA* gene is strongly repressed by the two-component ArcBA system. Due to the cAMP-CRP complex glucose can also prevent the transcription of the *dctA* gene. DcuAB and DcuC have similar functions as they catalyse the exchange, uptake and efflux of C4-dicarboxylates under anaerobic growth conditions (Zientz et al., 1999). DcuB and DcuC are the main transporters for succinate efflux during anaerobic growth (Zientz et al., 1999). While the *dcuA* gene is expressed constitutively, both DcuB and DcuC are activated by the O_2_-dependent regulator FNR. Furthermore, DcuB is repressed by nitrate due to the two-component NarXL regulatory system and activated by the two-component DcuSR system in response to presence of fumarate (Overton et al., 2006). CitT is a citrate:succinate antiporter, which is regulated by the two-component CitAB system in response to citrate (Scheu et al., 2012). Finally, the three proteins DctPQM in *Rhodobacter capsulatus* form a C4-dicarboxylate transporter which is in *Pseudomonas aeruginosa* dependent on the two-component dctSR system (Forward et al., 1997; Valentini et al., 2011).

*C. jejuni* contains all the enzymes for a complete oxidative TCA cycle, central to a flexible energy metabolism. *Campylobacter* possess only the C4-dicarboxylate carriers, DctA and DcuAB, some strains also contain one or two proteins similar to DcuC (Hofreuter et al., 2006). Like in other bacteria under oxygen-limited conditions, the transcription of the *C. jejuni dcuA* and *dcuB* genes is upregulated and under these conditions the antiporters are able to transport aspartate and fumarate (Woodall et al., 2005; Guccione et al., 2008). In contrast, all the transcription factors known to regulate the C4-dicarboxylate transporters carriers in other bacteria are lacking in *C. jejuni*.

Recently, we have shown that the two-component RacR/RacS system of *C. jejuni* directly represses the operon *aspA*-*dcuA*-*cj0089* under oxygen-limited conditions in the presence of nitrate (van der Stel et al., 2015). In this work, we investigated the function and regulation of all C4-dicarboxylate carriers in *C. jejuni*.

## Materials and methods

### Bacterial strains, media, and growth conditions

Bacterial strains and plasmids used in this study are listed in Table 1. *C. jejuni* was routinely cultured under microaerophilic conditions (5% O_2_, 10% CO_2_, 75% N_2_) on Blood Agar Base No. 2 (BA) medium containing 5% horse blood or in Heart Infusion broth (HI; Oxoid). Kanamycin (25 μg ml^−1^) and/or chloramphenicol (15 μg ml^−1^) and/or spectinomycin (30 μg ml^−1^) were added when appropriate. *E. coli* strains were routinely grown at 37°C in Luria–Bertani (LB) broth or on LB agar plates supplemented with ampicillin (50 μg ml^−1^), kanamycin (30 μg ml^−1^) or chloramphenicol (34 μg ml^−1^).

**Table 1:** **Bacterial strains and plasmids used in this study**.

**Strains or plasmids**	**Genotype or relevant characteristics**	**Source or reference**
**STRAINS**		
*C. jejuni* 81116	wildtype	Palmer et al., 1983
dctA	81116 derivative *dctA*::Cm	This study
dcuA	81116 derivative *dcuA*::Cm	This study
dcuB	81116 derivative *dcuB*::Km	This study
dcuC	81116 derivative *dcuC*::SpeC	This study
dcuC2	81116 derivative *dcuC2*::Cm	This study
dcuAB	81116 derivative *dcuA*::Cm *dcuB*::Km	This study
dcuABC	81116 derivative *dcuA*::Cm *dcuB*::Km, *dcuC*::SpeC	This study
*E. coli* PC2955	*relA1 Φ80dlacZΔM15 phoA8 hsdR17 recA1 endA1 gyrA96 thi-1 relA1 luxS glnV44*	NCCB
**PLASMIDS**		
pJet 1.2 blunt	Ap^R^ PCR cloning vector, Amp^r^	*Fermentas, thermoscientific*
pAV35	Ap^R^ Cm^R^ pBluescript II SK containing *Campylobacter coli* Cm^R^ cassette	van Vliet et al., 1998
pMW2	Ap^R^ Km^R^ pBluescript KS containing *C. jejuni* Km^r^ cassette	Wösten et al., 2010
pZW2	Ap^R^ Spec^R^ *E. coli-C. jejuni* shuttle vector	Zhou et al., 2012
pNBspec	Ap^R^ Spec^R^ pAV35 containing Spec^R^ cassette	This study
pJetdctA	Ap^R^; 6.2 kb pJet containing *dctA* region	This study
pJetdcuA	Ap^R^; 7.5 kb pJet containing *dcuA* region	This study
pJetdcuB	Ap^R^; 6.3 kb pJet containing *dcuB* region	This study
pJetdcuC	Ap^R^; 6.6 kb pJet containing *dcuC* region	This study
pJetdcuC2		
pJetdcuC2	Ap^R^; 8.7 kb pJet containing *dcuC2* region	This study
pJetdctA::Cm	Ap^R^ Cm^R^ pJet containing *dctA* gene on a 2009 bp fragment	This study
pJetdcuA::Cm	Ap^R^ Cm^R^; 5.9 kb, *dcuA* replaced by Cm^R^	This study
pJetdcuB::Km	Ap^R^ Km^R^; 6.5 kb, *dcuB* replaced by Km^R^	This study
pJetdcuC::Spec	Ap^R^ Spec^R^; 6.2 kb, *dcuC* replaced by Spec^R^	This study
pJetdcuC2::Cm	Ap^R^ Cm^R^; 8.5 Kb, *dcuC2* replaced by Cm^R^	This study

### Construction of a *dcuA, dcuB, dcuC, dcuC2* or *dctA* mutant

To disrupt the *dcuA, dcuB, dcuC, dcuC2*, or *dctA* genes, the genes as well as ~1 kb of the flanking regions were first amplified by PCR using the primer pairs dcuA-F/dcuA-R, dcuB-F/dcuB-R, dcuC-F/dcuC-R, dcuC-2F/dcuC-2R, or dctA-F/dctA-R, respectively. Primers are listed in Table 2. The ~3 kb PCR fragments were ligated into pJET1.2/blunt Cloning Vector resulting into the plasmids pJETdcuA, pJETdcuB, pJETdcuC, pJETdcuC2, and pJETdctA. Inverse PCR was performed on the plasmids pJETdcuA, pJETdcuB, pJETdcuC, and pJETdctA using the primers sets dcuABamHI F/dcuABamHI R, dcuBBamHI F/dcuBBamHI R, dcuCBamHI F/dcuCBamHI R, or dctABamHI F/dctABamHI R, respectively, to delete the *dcuA, dcuB, dcuC*, and *dctA* genes present in pJET and to introduce a *Bam*HI restriction site. The pJETdcuA and pJETdctA inverse PCR fragment were ligated to a *Bam*HI fragment containing a chloramphenicol resistance gene of pAV35 resulting in the knock-out constructs pJETdcuA::Cm and pJETdctA::Cm. The pJETdcuB and pJETdcuC inverse PCR fragment were ligated to a *Bam*HI fragment containing the kanamycin resistance gene of pMW2 or spectomycin resistance gene of pNBspec resulting into the knock-out constructs pJETdcuB::Km and pJETdcuC::speC, respectively. Plasmid pNBspec is a pAV35 derivate containing the spectomycin resistance gene of pZW2 (Zhou et al., 2012). pNBspec was constructed by amplifying pAV35 with the primers RBSCATrev and CATstop and the spectomycin resistance gene of pZW2 with the primers RBSspec and Specstop. In a second PCR these two PCR fragments were connected and after self-ligating of the PCR product pNBspec was obtained. To disrupt the *dcuC2* gene, plasmid pJETdcuC2 was digested with *XmaJI* and ligated to an *XbaI* fragment containing the pAV35 chloramphenicol resistance gene, resulting in the pJETdcuC2::Cm knock-out construct. To mutate the *dcuA, dcuB, dcuC, dcuC2, and dctA* genes, the knock-out constructs pJETdcuA::Cm, pJETdcuB::km, pJETdcuC::speC, pjetDcuC2::Cm, and pJETdctA::Cm were introduced by natural transformation in *C. jejuni* 81116. Homologous recombinations resulting in double cross-over events were verified by PCR.

**Table 2:** **Primers used in this study**.

**Primer**	**DNA sequence (5′-3′)**
**MUTANTS**	
dcuA-F	AGTCAAAGTACTAATGATGC
dcuB-R	TCAAGAGCTGCAACAGGACC
dcuA-BamHI F	AGGATCCTTACTTGCAAAATTATCATTATATCC
dcuA-BamHI R	AGGATCCGGCTTTGTATTAGCTCCTATGCTTATT
dcuB-F	TTAGATCCTGTAGGAGTGGG
dcuB-BamHI R	AGGATCCCTCACTAAGGCTTGTTAAAAAGTCC
dcuB-BamHI F	AGGATCCATTTTATTGCTATGGCAGCGGGTTAT
dcuC-F	TAAAATATTGATAATTCTTGTGAGT
dcuC-R	GCCTTTATTATCGGCGAAATTTGCA
dcuC-BamHI F	AGGATCCAAATAGCAGTATCTTTAAATAATAAATA
dcuC-BamHI R	AGGATCCGCTAAAAGTGTAGAACATATCATAAA
dcuC-2F	TAAAATATCACCCATATCTCCAATTTT
dcuC-2R	TATCCACCCCAGCTAGCACCAATTGA
dctA-F	CATAAATTTACCCCTTTTTATTGAAA
dctA-R	CCCTTTTTTATTTTAATTATACACTTA
dctA-BamHI F	TGGATCCTTGCCATCTGGGATAAACAAATTGAT
dctA-BamHI R	TGGATCCAGCAATGGCTAATTCTTTATCTATATA
**SPEC CASSETTE**	
RBSCATrev	TTATCCTCCGTAAATTCCGATTTGTTG
CATstop	TAAAATCCCAGTTTGTCGCACTG
RBSspec	CGGAATTTACGGAGGATAAATGATGAATAGTTATGAAGTAAC
Specstop	GCGACAAACTGGGATTTTAAGCAAAACCTTTTATTTTTTGTTGAAGG
**RT-PCR**	
dctARtaq	GTGTAACTGGATCAGGTTTTATAGTGCTT
dctAFtaq	GCTAAGGTTGCATTTGCTTCTGAA
dcuBFtaq	TGGACTTATGCTGTAATGCTTCTTTTA
dcuBRtaq	GCCAAAGGAACAAAAGCTGAA
aspAFtaq81116	TTTGTTAGAGCTTTGGCTAGAGTAAAAA
aspARtaq81116	CGCTTTAATAATCGCATCTTGGA
gyrAFtaq81116	ACGACTTACACGACCGATTTCA
gyrARtaq81116	ATGCTCTTTGCAGTAACCAAAAAA
dcuCFtaq	CACTTGGTGGAGTTAATATCCTTGCT
dcuCRtaq	AAAGAATTCCAAACCATGCAAAA
dcuC2Ftaq	TGCTTGCAGTTTGTGCTTTCTT
dcuC2Rtaq	TTGGGATAAGTGGAGCAAAGCT
dcuAFtaq	GTTTCGGCACTTTTTGTGCTT
dcuARtaq	CCAATTCTAGTCGTTCCTGTATCATC
**EMSA**	
dcuBR	ATTTGGATTGCAAATTGCCCT
dcuBF	AAAATTCTATCAATCTATCAAACC
dcuCR	TAAGTATATAATAAGCAACGACAATT
dcuCF	TTTCAGTAACCAAACTATACATATT
dctAF	GCGGTTTTCTTTCAGCTAAAGTTTGA
dctAR	GCAAATAATGAGAAATTTTGTAACATT
dcuC2R	GCTGAGTATGGACCAATGGCCTTTG
dcuC2F	TACTCTTTATACTTTAAAACATTTCTT
aspAF	AGCTTGCAAAAATATATTAATTT
aspAR	TAATAAACCTCATCAGAGATTTC

### RNA isolation

RNA was extracted from *C. jejuni* wild-type grown in HI with or without 25 mM serine, aspartate, fumarate or succinate under 10 or 0.3% oxygen concentration at logarithmic (10 h) or stationary phase (20 h). RNA was also extracted from the wild-type, the *racR* mutant and the *racR* complemented strain grown in HI with 50 mM of NaNO_3_ under 0.3% oxygen concentration until late logarithmic (log) phase (16 h) using the RNA-Bee™ kit (Tel-Test, Inc.). RNA samples were treated with RNAse-free DNAse I (Fermentas) according to the manufacturer's manual.

### Real-time RT-PCR

Real-time RT-PCR analysis was performed as previously described (Wösten et al., 2004). Primers used in this assay are listed in Table 2. The calculated threshold cycle (Ct) for each gene amplification was normalized to the Ct of the *gyrA* gene amplified from the corresponding sample before calculating fold change using the arithmetic formula 2^−ΔΔCt^ (Schmittgen, 2001). Each sample was examined in four replicates and was repeated with at least two independent preparations of RNA. Standard deviations were calculated and are displayed as error bars.

### Electrophoretic mobility shift assay (EMSA)

Recombinant His-tag labeled RacR was isolated and EMSA were performed as described before (van der Stel et al., 2015). The promoter regions upstream of the genes *aspA, dcuB, dcuC, dcuC2*, and *dctA* were amplified by PCR using the primer pairs listed in Table 2 and *C. jejuni* 81116 genomic DNA as template. Radioactive labeled PCR products, ~25 pmol, were incubated with 0 or 50 pmol of recombinant RacR and 25 pmol RacScyto (van der Stel et al., 2015) for 20 min at RT in binding buffer containing 20 mM Tris, pH 7.4, 5 mM MgCl_2_, 50 mM KCl, 2 mM ATP, 50 μg/ml bovine serum albumin, 10 μg/ml poly-(dI-dC), and 10% glycerol. For competition assays RacR was pre-incubated for 15 min with 10 times excess of unlabelled PCR fragment. Samples were run on 6% non-denaturing Tris-glycine polyacrylamide gels at 4°C. After electrophoresis, gels were dried and autoradiographed.

### Growth experiments

Growth curves were generated under microaerobic conditions (10% O_2_, 10% CO_2_, 70% N_2_, 10% H_2_) or under oxygen-limited conditions (0.3% O_2_, 10% CO_2_, 79% N_2_, 10% H_2_) at 42°C in a “honeycomb” 10 × 10 well micro-plate using a Bioscreen C MRB (Oy Growth Curves Ab Ltd) computer-controlled incubator placed in the anaerobic chamber (Coy labs, Michigan, United States). *Campylobacter* precultures grown for 6 h in HI were diluted to OD_600nm_ of 0.01 in fresh HI media containing 25 mM fumarate, 25 mM aspartate, 25 mM serine, or 25 mM succinate with or without 25 mM nitrate. The optical density at 600 nm was measured every 15 min. The experiments were repeated at least three times in duplicate.

### High-performance liquid chromatography (HPLC)-MS-MS analysis

During the growth experiments of *C. jejuni* at 10% O_2_, culture samples (50 μl) were taken at 4, 8, 16, and 24 h, under oxygen-limited conditions (0.3% O_2_) samples were taken at 6, 12, 24, and 36 h. The culture samples were centrifuged at 14,000 rpm for 5 min. The supernatants were diluted in milli-Q water and adjusted to pH 2.4 with formic acid and injected on a Synergi 4u Fusion-RP (150 × 2.0 mm, particle size of 4 μm) analytical column (Phenomenex, Utrecht, NL). Elution was performed isocratically with milli-Q (adjusted to pH 2.4 with formic-acid): acetonitrile (9:1 [v/v]) at a flow rate of 0.3 ml/min, and the column effluent was introduced by an atmospheric pressure chemical ionization (APCI) interface, in negative mode with an ionization current of −1 μA and a source temperature of 350°C, into a 2000 QTRAP mass spectrometer (Sciex, Toronto, ON). For maximal sensitivity and for linearity of the response, the mass spectrometer was operated in multiple-reaction monitoring (MRM) mode at unit mass resolution. Peaks were identified by comparison of retention time and mass spectrum with authentic standards. Ion transitions monitored were *m/z* 115.0/71.0 (fumarate), 117.0/73.0 (succinate), 132.1/88.0 (aspartic acid), and 104.0/74.0 (serine) at collision energies of −12, −15, −20, and −18V, respectively. Simultaneously the four molecules were monitored by single-ion monitoring (SIM). Data were analyzed with Analyst software version 1.6.1 (Applied Biosystems).

### Statistical analysis

Prism software (GraphPad, San Diego, CA) was used for statistical analysis. Data was expressed as mean ± *SD*. Results were analyzed by two tailed paired *t*-test, *p* < 0.05 was considered statistically significant.

## Results

### Genome locations of the *C. jejuni* C4-dicarboxylate transporters

**Figure 1:**
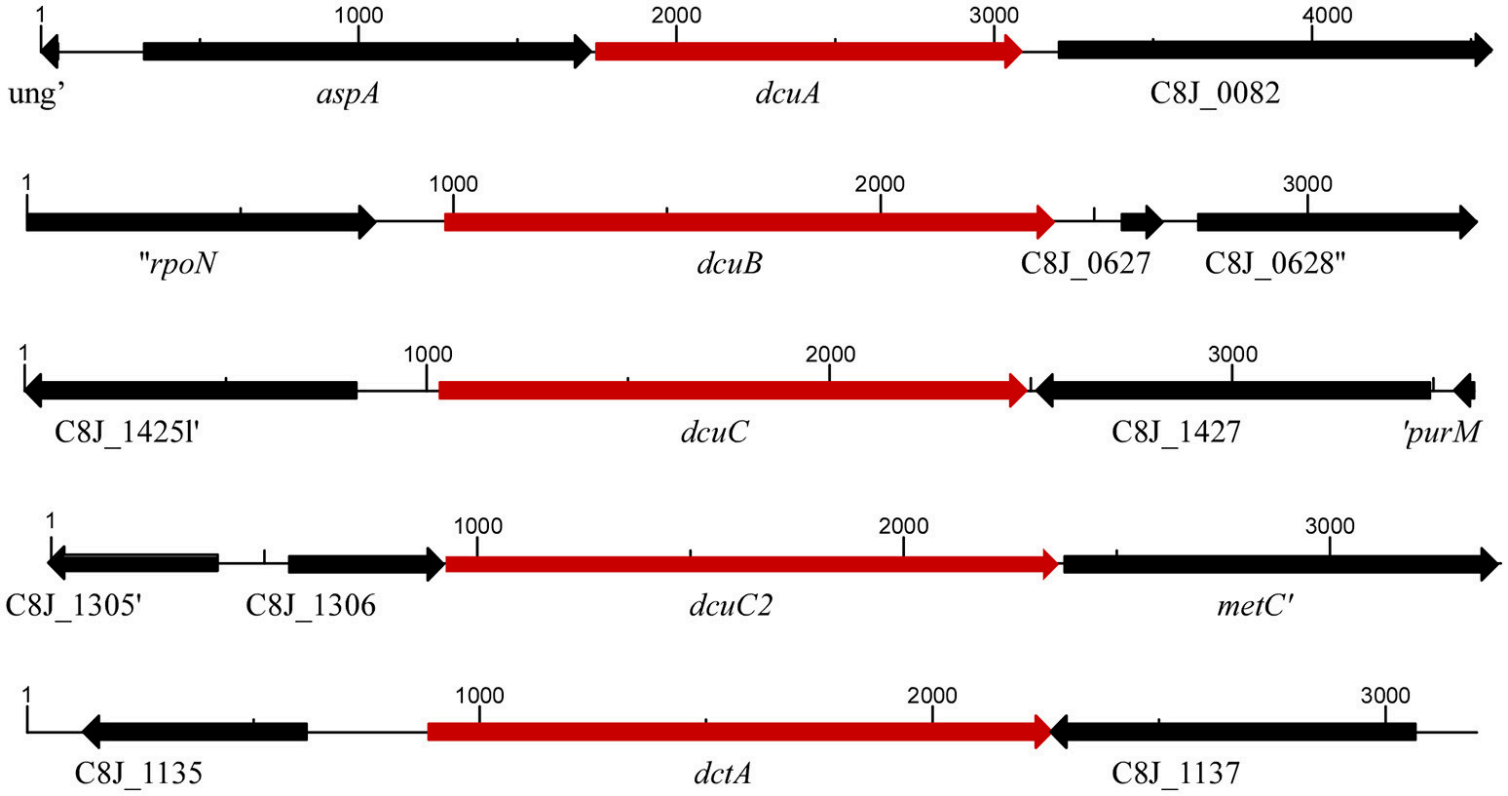
**Organization of the C4-dicarboxylate transporters in the genome of *C. jejuni* 81116**. The size and orientation of the genes in C4 transporters regions are indicated.

Genome analysis revealed that *C. jejuni* possesses up to five putative C4-dicarboxylate transporters, DctA, DcuA, DcuB, and some strains contain one or two homologs of the DcuC C4-dicarboxylate transporter. *C. jejuni* 81116 possess five C4-dicarboxylate transporters, which are all located at a different locus in the genome (Figure 1). The genes *dcuB, dcuC*, and *dctA* are not co-transcribed with other genes located in a single-gene operon, while *dcuA* is located in one operon together with the *aspA* gene and the *dcuC2* gene is located in one operon with C8J_1306 and the *metC* genes.

### Transcription regulation of the *C. jejuni* C4-dicarboxylate transporters

**Figure 2:**
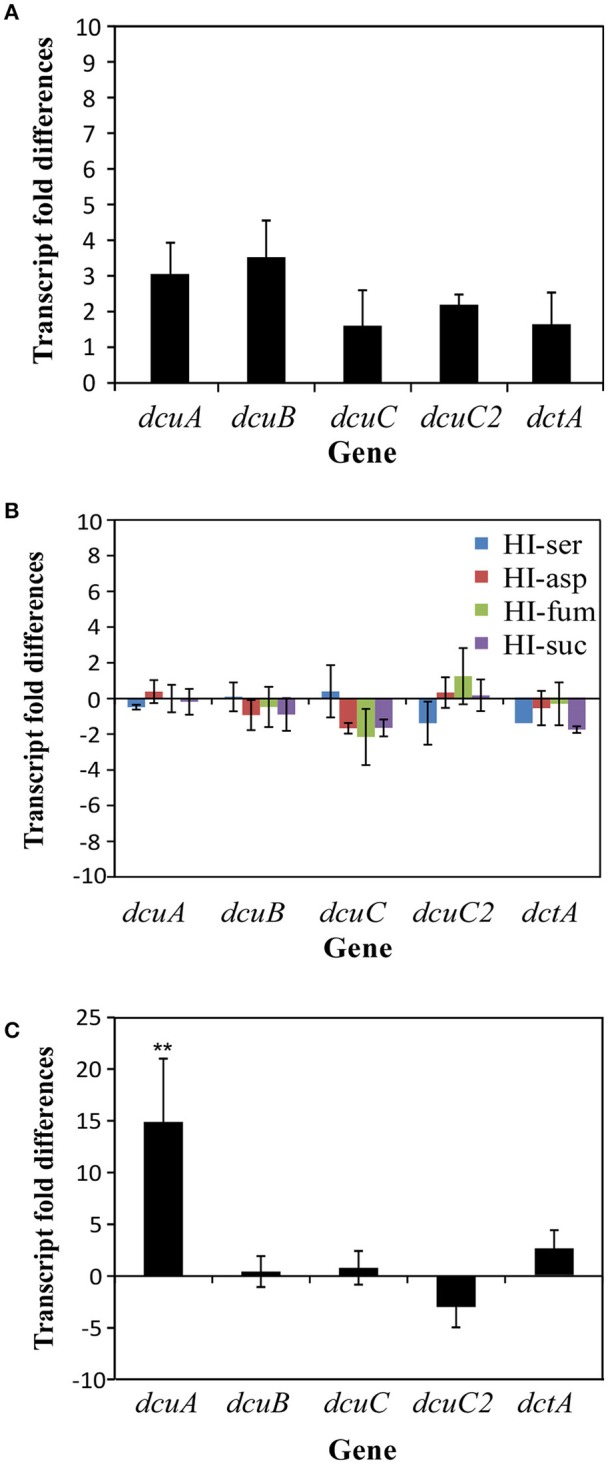
**Influence of oxygen, carbon sources, and growth phase on the transcription of the C4-dicarboxylate transporter genes as measured by real-time RT-PCR. (A)** Transcript fold difference of the *dcuA, dcuB, dcuC, dcuC2*, and *dctA* genes in logarithmic phase grown wild-type *C. jejuni* (10 h) at 10% O_2_ compared to 16 h at 0.3% O_2_. **(B)** The effect of adding various carbon sources to the HI culture medium on the transcription of the C4-dicarboxylate genes. Hereto total RNA was isolated from wild-type grown in HI or HI with 25 mM serine, aspartate, fumarate, or succinate for 10 h. **(C)** Influence of the growth phase on the transcription of the C4-dicarboxylate genes as estimated for wild-type bacteria by real-time RT-PCR. Total RNA was extracted from logarithmic (10 h), or stationary (20 h) phase cultures. Fold change relative to the transcription levels was calculated using the arithmetic formula (2^−ΔΔCt^). The *gyrA* gene was used as normalization gene. Data of four independent experiments with two independent preparations of RNA are presented as mean values ± standard deviation, ^**^P < 0.01.

In a number of bacterial species, the C4-dicarboxylate transporters are regulated by oxygen, the available C4-dicarbolytes and/or by growth phase. To investigate whether this also applies for the C4-dicarboxylate transporters in *C. jejuni* we performed real-time RT PCR. We first used mRNA isolated from the wild-type 81116 strain grown at 10 or 0.3% oxygen (Figure 2A). Only the transcripts of the *dcuA* and *dcuB* genes showed a minor increase of three- to four-fold under oxygen limited conditions. Addition of serine or the C4-carbon sources, aspartate, succinate or fumarate to the culture medium of the wild-type grown until the logarithmic or stationary phase (data not shown) at 0.3 or 10% oxygen (data not shown) did not influence the transcription of the C4-dicarboxylate transporters (Figure 2B). Finally, we tested whether the growth phase (by comparing logarithmic vs. stationary phase) influences the transcription of the C4-dicarboxylate genes (Figure 2C). Only the transcription of the *dcuA* gene was regulated by growth phase as a 15-fold higher transcript level was observed at the logarithmic phase compared to the stationary phase. These results indicate that the regulation of C4-dicarboxylate transporters in other bacteria cannot be extrapolated to that of *C. jejuni*.

### *dcuA* and *dcuB* are directly regulated by *racR*

**Figure 3:**
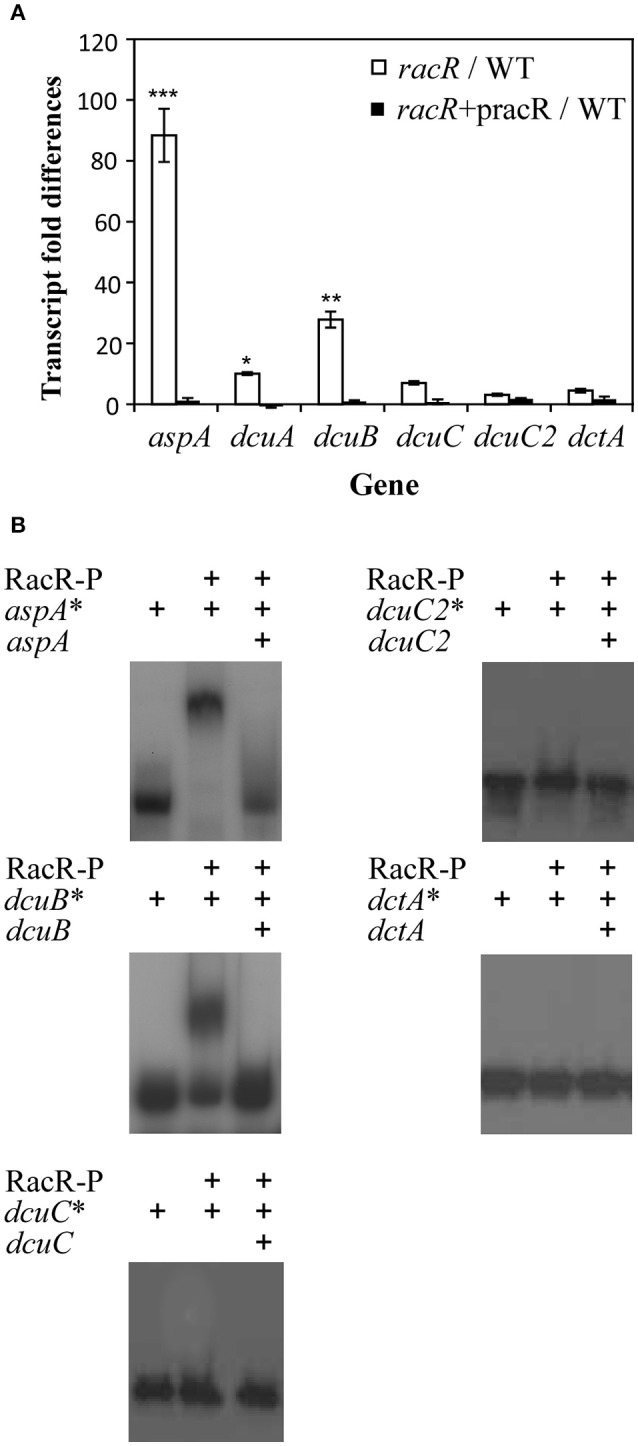
**RacR directly regulates the *dcuA* and *dcuB* promoters. (A)** Real-time RT-PCR data of the *dcuA, dcuB, dcuC, dcuC2*, and *dctA* genes in wild-type *C. jejuni* compared to the RacR mutant or the complemented RacR mutant strain. Data of four independent experiments with two independent preparations of RNA are presented as mean ds ± standard deviation, ^*^P < 0.05, ^**^P < 0.01, ^***^P < 0.001. **(B)** Electrophoretic mobility shift assays with the *aspA, dcuB, dcuC, dcuC2*, and *dctA* promoter regions labeled with [γ-^32^P]ATP and phosphorylated RacR protein. Radioactive labeled promoter regions are marked with ^*^. RacR was phosphorylated by RacScyto in the presence of ATP. The specificity of the protein-DNA interaction was determined by the addition of a 10-fold excess of unlabelled promoter region DNA.

We previously showed that the two-component regulator RacR regulates the *dcuA* transporter gene. As *dcuC* and *dcuC2* were not present on the used microarrays slides we performed real-time RT PCR using RNA isolated of the wild-type and RacR mutant grown under RacR inducing conditions (0.3% O_2_ and 25 mM nitrate). Transcription of *dcuC, dcuC2*, and *dctA* genes was not affected by mutation of *racR*, however like the proven RacR dependent genes *dcuA* and *aspA*, a decrease of the *dcuB* transcription was observed in the *racR* mutant (Figure 3A).

To investigate whether RacR directly regulates the *dcuB* promoter we performed electrophoretic mobility shift assays (EMSA). As previously shown, phosphorylated RacR was able to bind to the ^32^P-labeled *aspA*-*dcuA* promoter region, however RacR also binds to the *dcuB* promoter (Figure 3B). The band shift disappeared when unlabelled DNA corresponding to the *dcuB* promoter region was added in excess. As expected from the real-time RT PCR results, the *dcuC, dcuC2*, and *dctA* promoter regions are not recognized by the RacR protein. These results show that the *dcuA* as well as *dcuB* are directly regulated by the two-component system regulator RacR.

### Dcta is active at elevated oxygen levels

**Figure 4:**
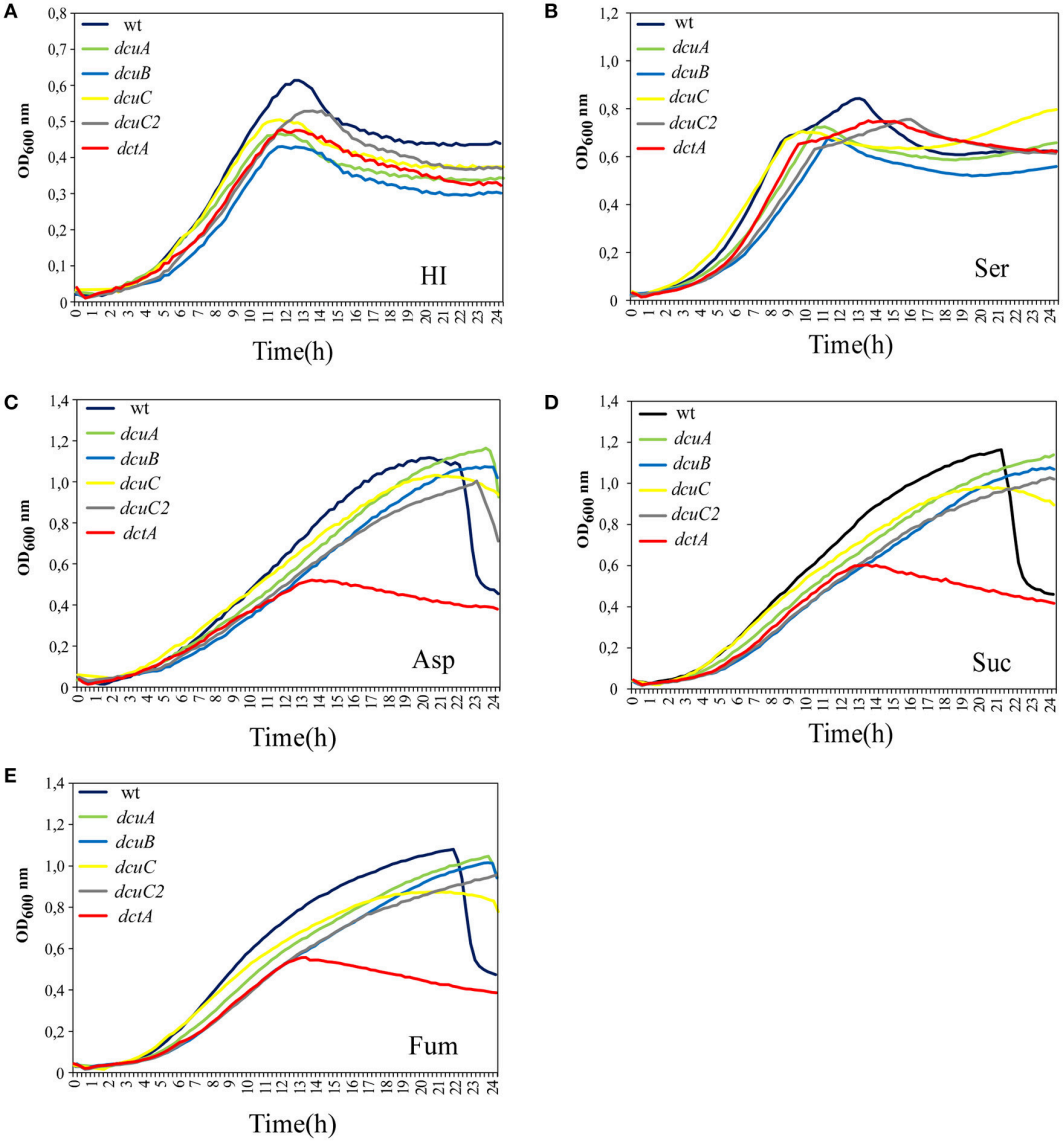
**DctA is the only C4-carboxylate transporter necessary at high oxygen conditions (10% O_2_)**. Growth curves of *C. jejuni* wild-type or the *dcuA, dcuB, dcuC, dcuC2*, and *dctA* mutants were generated in HI **(A)**, or HI with the addition of 25 mM serine **(B)**, aspartate **(C)**, succinate **(D)**, or fumarate **(E)** under microaerobic conditions (10% O_2_, 10% CO_2_, 70% N_2_, and 10% H_2_) at 42°C. The optical density at 600 nm was measured every 15 min. The experiments were repeated three times in duplicate.

To phenotypically address the function of the C4-dicarboxylate transporter in *C. jejuni* we disrupted the *dctA, dcuA, dcuB, dcuC*, and *dcuC2* genes by substituting large parts of the genes with an antibiotic resistance cassette. Although the transcription of C4-dicarboxylate transporter in *C. jejuni* is not regulated by oxygen (Figure 2A), we investigated whether oxygen has an influence of the activity of these transporters. Hereto growth curves in the presence of 10% O_2_ were generated of the wild-type and the *dctA, dcuA, dcuB, dcuC*, and *dcuC2* mutants in HI or with the addition of 25 mM serine, aspartate, succinate, or fumarate. No clear growth differences between the strains were observed when they were growing in HI or HI with 25 mM serine (Figures 4A,B). However, all strains reached a higher OD when serine was added to the medium, suggesting that the available serine or carbon is a limited compound in HI. When one of the C4-carbon sources, aspartate, succinate, or fumarate were added to the HI medium, the maximum final OD of all strains except for the *dctA* mutant, was also higher compared to HI alone (Figures 4C–E), indicating that carbon availability in HI is a limiting growth factor. The *dctA* mutant reached a similar maximum final OD in HI as in HI with additional aspartate, succinate or fumarate indicating that the *dctA* mutant is unable to utilize or to take up these carbon sources from the medium. Based on these results we conclude that the DctA transporter is the only C4-dicarboxylate transporter needed to allow *C. jejuni* to growth on C4-carbon sources at high oxygen levels.

### Main function of *dctA* is the uptake of succinate from the medium at elevated oxygen levels

**Figure 5:**
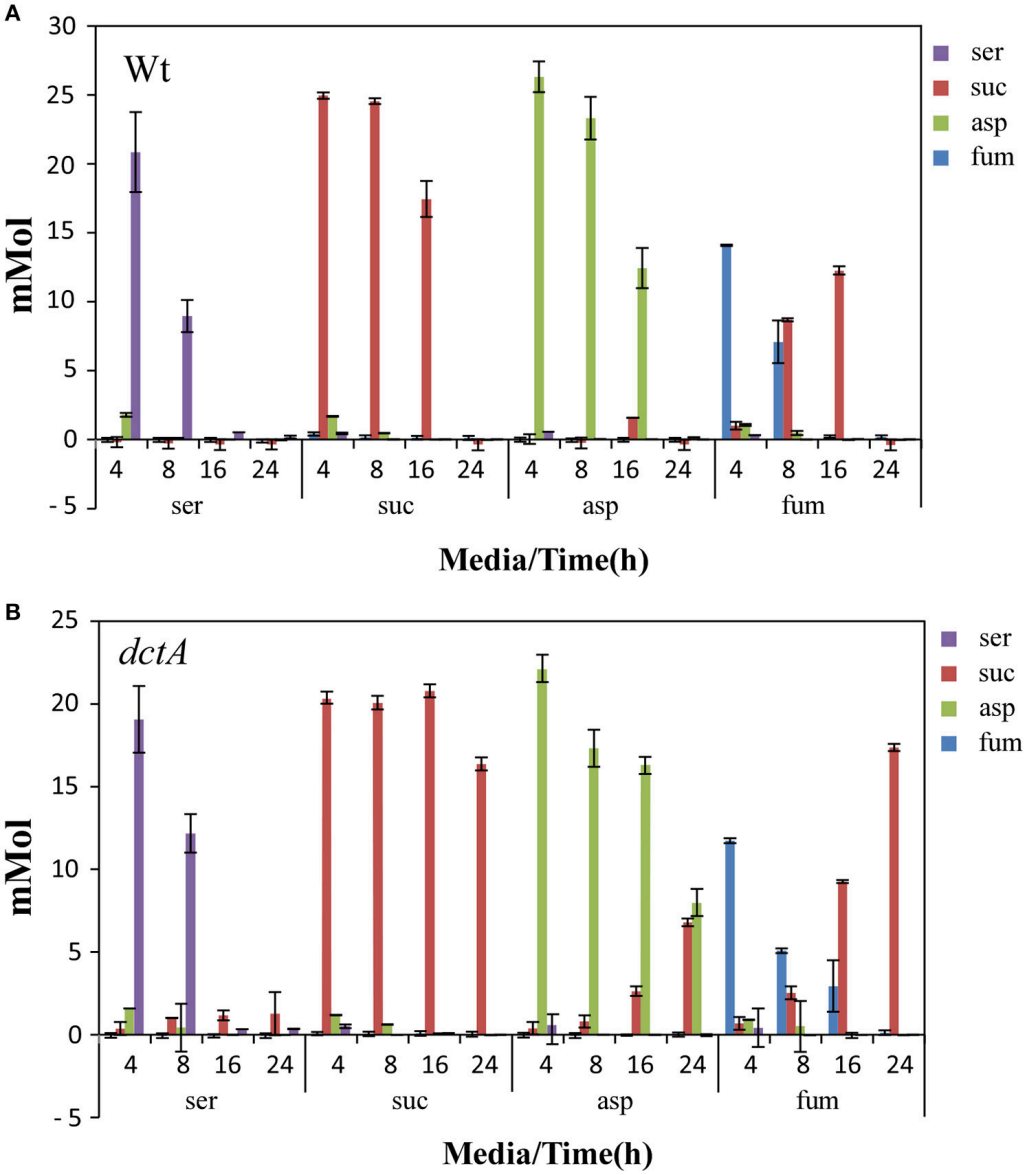
**DctA transports succinate into the cell**. HPLC-MS-MS analysis of the culture supernatants of wild-type **(A)** and *dctA* mutant **(B)** taken after 4, 8, 16, or 24 h of growth under 10% O_2_ with 25 mM serine, aspartate, fumarate, or succinate. Data represent the mean and standard error of three independent experiments.

To investigate why the *dctA* mutant is unable to use aspartate, fumarate, and succinate at 10% O_2_, we measured concentrations of serine, aspartate, succinate, and fumarate in the culture supernatants of the wild-type and C4-dicarboxylate transporter mutants at 4, 8, 16, and 24 h (Figure 5A). Without the addition of extra C4-dicarboxylate compounds HI medium contains no detectable fumarate, 0.4 mM succinate, 1.0 mM aspartate, and 1.2 mM serine. All added carbon sources, serine, succinate, aspartate, and fumarate were completely utilized by the wild-type bacteria within 24 h growth at 10% O_2_, however serine and fumarate were removed earlier from the media than succinate and aspartate. Fumarate was converted to succinate and then secreted to the medium. Once the fumarate was completely used the secreted succinate was taken up again and utilized by *C. jejuni* (Figure 5A) Similar results were obtained for the *dcuA, dcuB, dcuC*, and *dcuC2* mutants (data not shown). A different result was obtained for the *dctA* mutant. The *dctA* mutant was still able to take up serine, to convert fumarate to succinate and, although reduced, to take up aspartate (Figure 5B), but was unable to take up succinate from the media and accumulated in the supernatant of media containing excess serine, aspartate, or fumarate. These results indicate that the DctA transporter of *C. jejuni* is involved in the uptake of aspartate and indispensable is for the uptake of succinate from the media at elevated oxygen conditions.

### Redundancy of C4-dicarboxylate transporter function under oxygen-limited conditions

**Figure 6:**
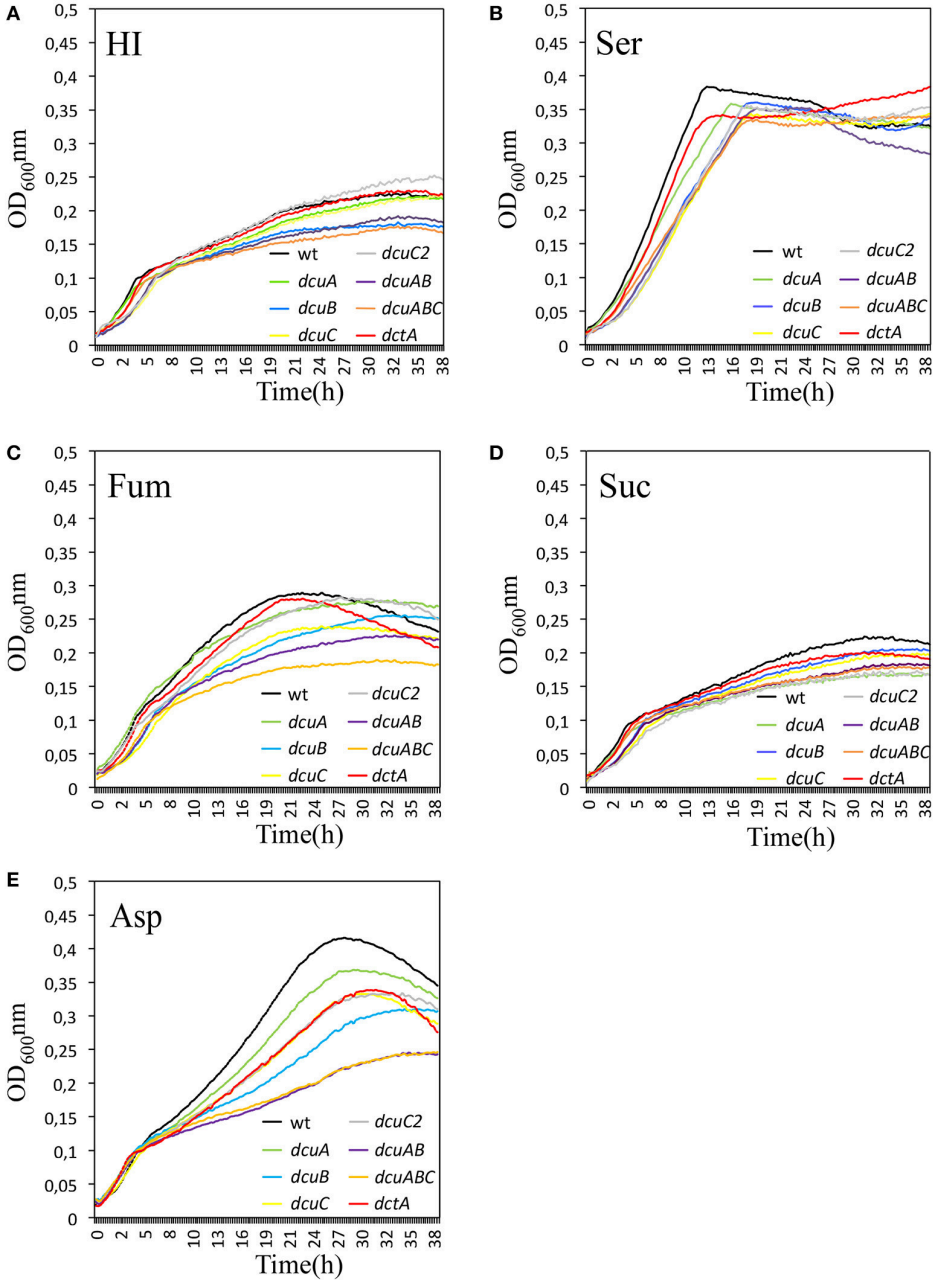
**DcuA and DcuB are the main C4-dicarboxylate transporters under oxygen-limited conditions**. Growth curves were generated of *C. jejuni* wild-type, the single mutants *dcuA, dcuB, dcuC, dcuC2*, and *dctA*, the double mutant *dcuA/B* and the triple mutant *dcuA/B/C* under oxygen-limited conditions (0.3% O_2_). Strains were growing in HI **(A)**, or HI with the addition of 25 mM serine **(B)**, fumarate **(C)**, succinate **(D)**, or aspartate **(E)** under oxygen-limited conditions (0.3% O_2_, 10% CO_2_, 79% N_2_, 10% H_2_) at 42°C. The optical density at 600 nm was measured every 15 min. The experiments were repeated three times in duplicate.

To investigate which of the C4-dicarboxylate transporters are active under oxygen-limited conditions, we generated growth curves of the wild-type, the *dctA, dcuA, dcuB, dcuC*, and *dcuC2* mutants as well as a double *dcuA*/*dcuB* and a triple *dcuA*/*dcuB*/*dcuC* mutant in HI or HI with the addition of 25 mM serine, aspartate, succinate, or fumarate (Figures 6A–E). The maximum optical densities reached under these oxygen-limited conditions were all lower compared to the growth curves obtained at 10% O_2_. No obvious growth differences were observed between the strains when they were grown in HI, HI + serine, or HI + succinate. The maximum OD of all strains was higher in HI + serine compared to HI alone, indicating that serine is utilized by *C. jejuni* under these conditions (Figure 6B). In contrast, succinate is not utilized under the oxygen-limited conditions as the growth curves generated in HI + succinate were similar as in HI. Growth defects were observed when fumarate or aspartate was added to the HI medium (Figures 6C,E). All single mutants grew less compared to the wild-type in HI + aspartate, especially the *dcuB* mutant. To investigate whether the C4-carboxylate transporter can replace each other function, we also tested double and triple mutants. A more severe growth defect in HI + aspartate compared to the *dcuB* mutant was observed for the *dcuA*/*dcuB* mutant but not for a *dctA*/*dcuA* (data not shown). A similar *dcuA*/*dcuB* growth defect was seen for the *dcuA*/*dcuB*/*dcuC* triple mutant, suggesting that *dcuC* is not involved in the utilization of aspartate. A small reduction in growth yield was observed for the single mutants *dcuC* and *dcuB* in HI + fumarate. The growth defect was more obvious in the triple mutant *dcuA/dcuB/dcuC*, suggesting that both DcuB and DcuC are involved in the utilization of fumarate.

### Secretion of succinate is dependent on a functional *dcuB* under limited oxygen conditions

**Figure 7:**
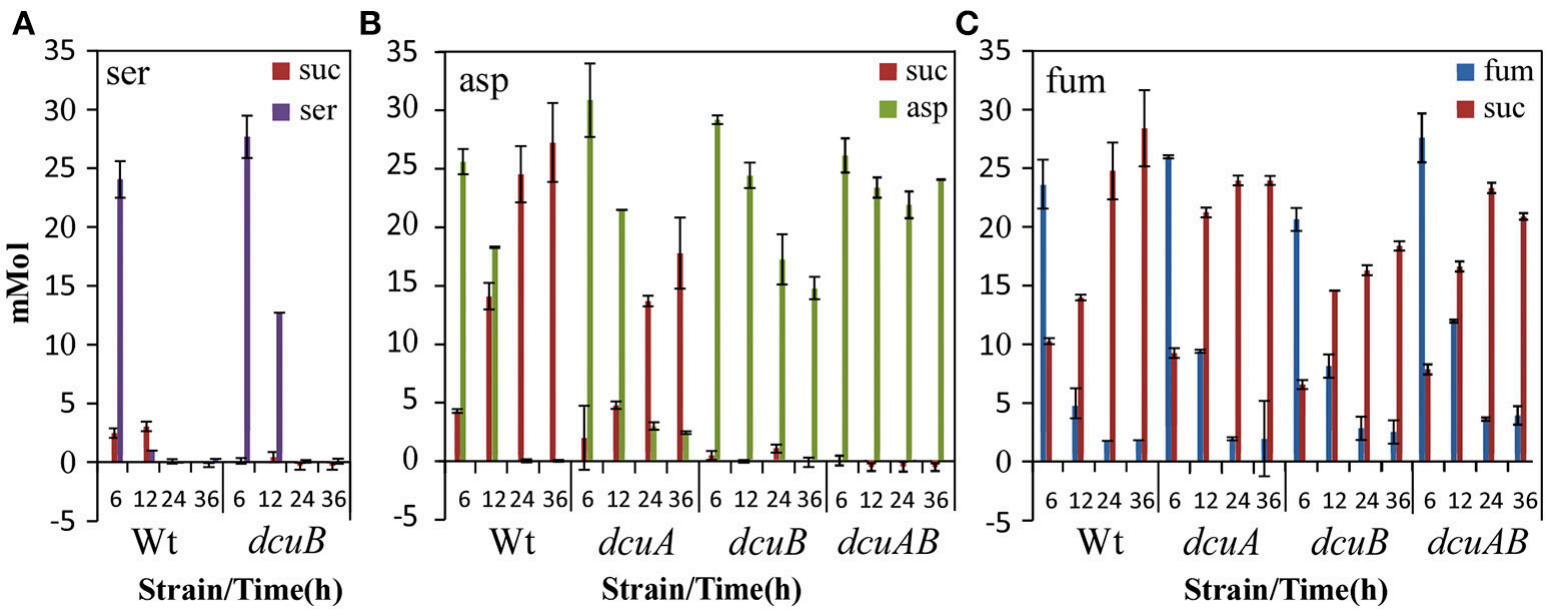
**DcuB secretes succinate into the environment under oxygen-limited conditions**. HPLC-MS-MS data of the supernatants of wild-type, *dcuA, dcuB*, or *dcuAB* mutant cultures taken after 6, 12, 24, or 36 h of growth at 0.3% O_2_ with 25 mM serine **(A)**, aspartate **(B)**, or fumarate **(C)**. Data represent the mean and standard error of three independent experiments.

To investigate the role of various active C4-transporters under oxygen-limited conditions we measured at time points 6, 12, 24, and 36 h the serine, aspartate, succinate, and fumarate concentration in the culture supernatants of the wild-type and C4-transporters mutants (Figure 7). Serine was used within 12 h by the wild-type and within 24 h by the *dcuB* mutant, the slowest grower in HI + serine (Figure 7A). When the strains were grown on aspartate they secreted succinate to the medium, except for the strains which were also mutated in the *dcuB* gene (Figure 7B). No succinate could be measured in the supernatant of the *dcuB* mutants showing that DcuB under oxygen-limited conditions transports succinate into the environment. Both the *dcuA* and *dcuB* mutants were able to utilize aspartate, however when both genes were mutated no decrease in the aspartate concentration in the medium was observed, indicating that both genes are needed to sequester aspartate from the medium. Growing the strains in excess fumarate revealed that not *dcuC*, but both *dcuA* and *dcuB* might be involved in the uptake of fumarate, as at 12 h the supernatant of these single mutants contain more fumarate than the supernatant of the wild-type (Figure 7C). The supernatant of all strains tested grown in excess fumarate contained succinate. However, the supernatant of the *dcuB* mutant strains contained less succinate, indicating that *Campylobacter* possesses a *dcu*-independent fumarate–succinate reductase.

### The availability of nitrate under oxygen-limited conditions reduces the role of the C4-dicarboxylate transporters

**Figure 8:**
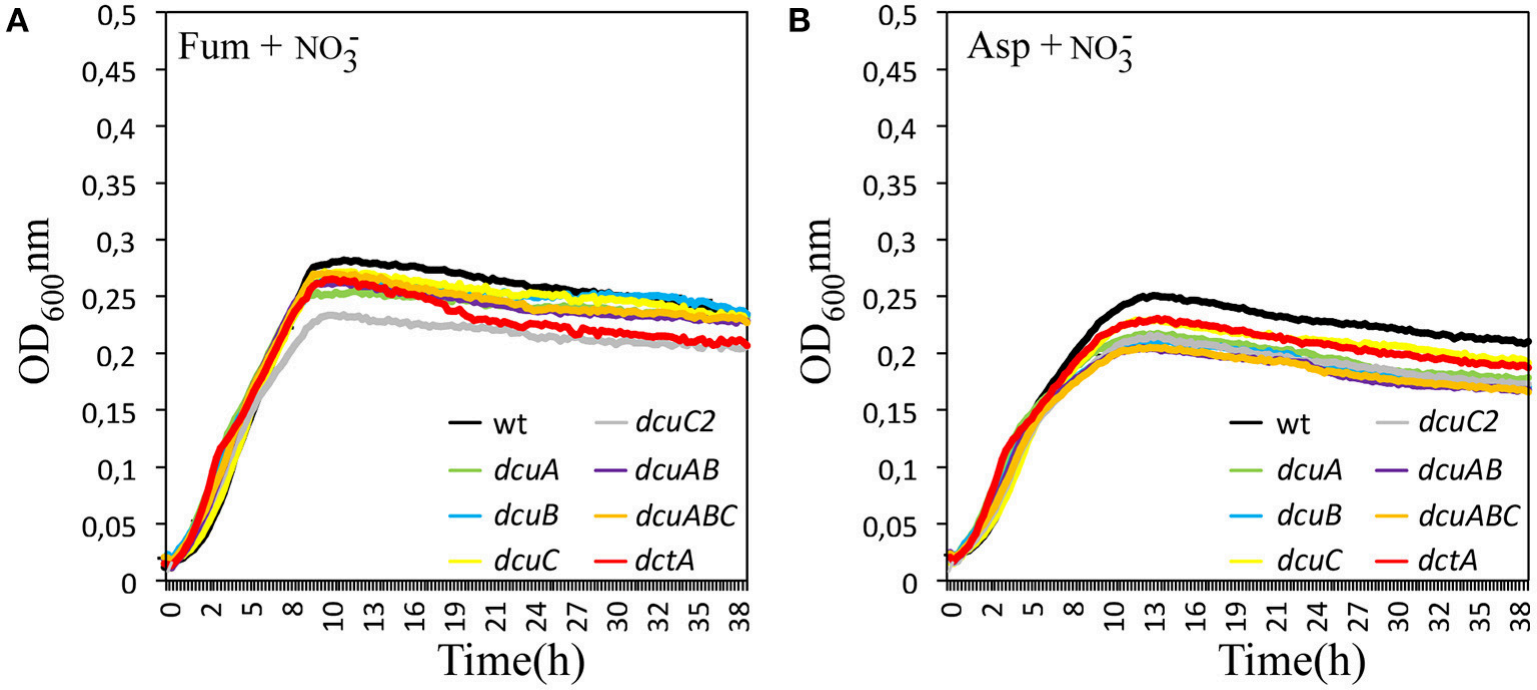
**Addition of nitrate under limited oxygen conditions nullifies the C4-dicarboxylate mutant phenotype**. Growth curves were generated of *C. jejuni* wild-type, the single mutants *dcuA, dcuB, dcuC, dcuC2*, and *dctA*, the double mutant *dcuA/B* and the triple mutant *dcuA/B/C* in HI with 25 mM nitrate and 25 mM fumarate **(A)** or aspartate **(B)** at 42°C under oxygen-limited conditions. The optical density at 600 nm was measured every 15 min. The experiments were repeated three times in duplicate.

We showed that RacR directly regulates by inhibition, the *dcuA* and *dcuB* genes under limited oxygen and high nitrate conditions. To address the role of the *dcu* genes under these conditions, we performed growth curves in HI under oxygen-limited conditions with nitrate and aspartate or fumarate (Figure 8). The growth rate as well as the maximum optical densities of the wild-type and the mutants were similar under these conditions, suggesting that the C4-dicarboxylate transporters are less active under these conditions.

### Nitrate prevents the secretion of succinate under oxygen-limited conditions

**Figure 9:**
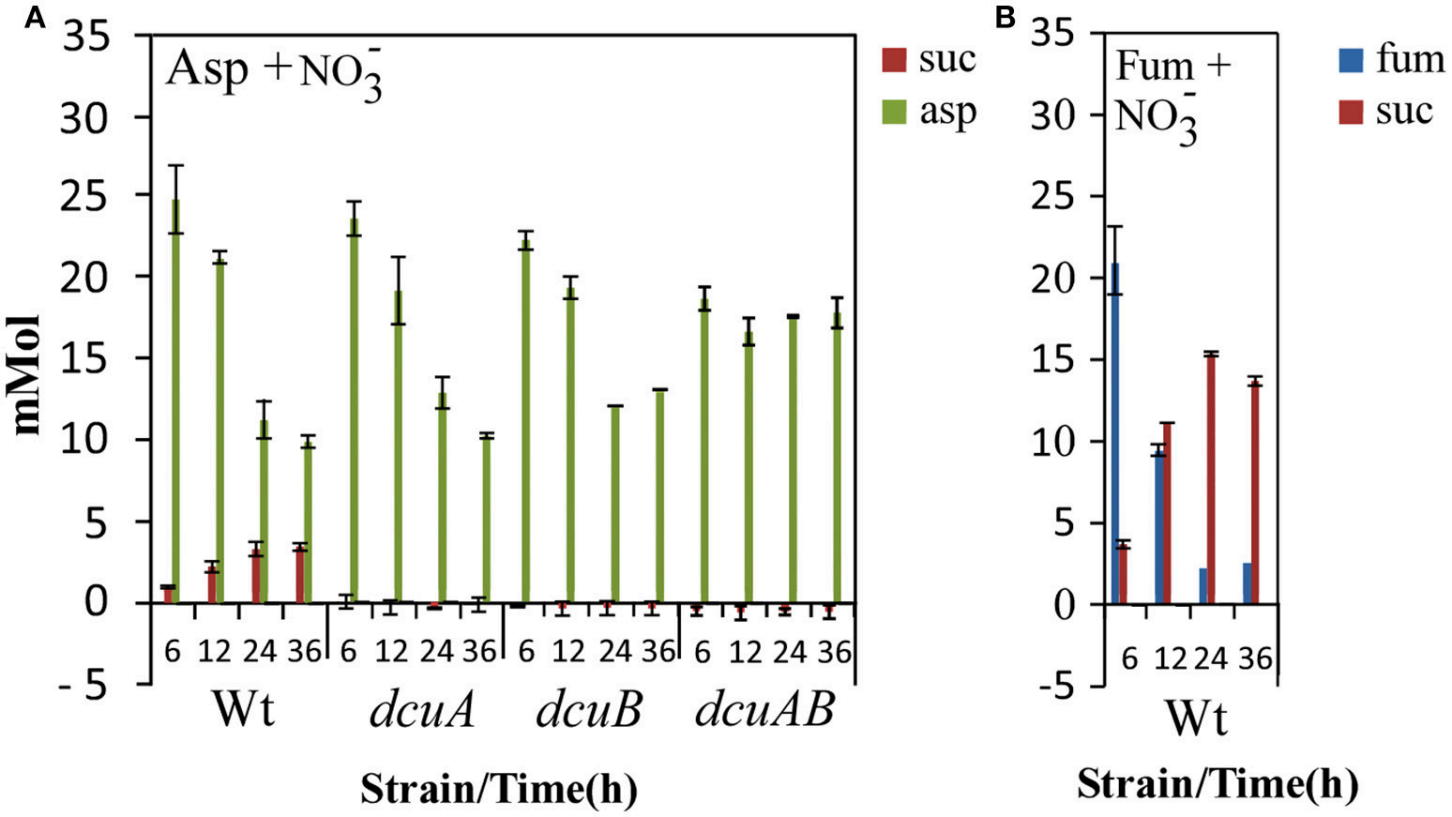
**Nitrate prevents the secretion of succinate to the environment**. HPLC-MS-MS of the supernatants of wild-type, *dcuA, dcuB*, or *dcuAB* mutant cultures taken after 6, 12, 24, or 36 h of growth under 0.3% O_2_ with 25 mM nitrate and 25 mM aspartate **(A)** or 25 mM nitrate and 25 mN fumarate **(B)**. Data represent the mean and standard error of three independent experiments.

To further address the role of the C4-dicarboxylate transporters under RacR inducing conditions, we measured the aspartate, fumarate, and succinate concentrations in the supernatant of the wild-type and the C4-dicarboxylate mutants at 6, 12, 24, and 36 h. The C4-carbon content in the supernatants of the strains growing under oxygen-limited conditions with nitrate were much more similar compared to the strains grown under oxygen-limited conditions (Figure 9). The aspartate uptake in the wild-type, *dcuA* and *dcuB* mutants under these conditions were similar, however only half of the aspartate in the supernatant of these cultures was utilized (Figure 9A). These results are in accordance with the growth curves and show that the C4-transporters are less active under these conditions. No reduction of the aspartate concentration was seen in the supernatant of the *dcuAB* mutant, confirming that both DcuA and DcuB are needed to take up aspartate from the medium. The amount of succinate produced by the wild-type under these conditions was nine-fold lower than under oxygen-limited conditions, similar results were seen for the *dctA* and *dcuC2* mutants. In all other mutants no succinate could be detected in the supernatant. These results show that the activity like the transcription (Figure 3A) of aspartate–fumarate–succinate pathway under oxygen-limited condition is strongly reduced when nitrate is available. In the wild-type the utilization of fumarate in the presence of nitrate was similar as without nitrate, however the amount of secreted succinate was two-fold lower when nitrate was present (Figure 9B). Similar results were obtained for all other mutants (data not shown), indicating that *Campylobacter* possesses a *dcu* nitrate independent fumarate–succinate reductase.

## Discussion

Dependent on the oxygen concentration, C4-dicarboxylate transporters have been shown to play an important role in the transport of C4-carbon sources such as fumarate, succinate, and aspartate. These carbon sources are even more important for the microaerophilic bacterium *C. jejuni* as most strains cannot ferment nor oxidize carbohydrates. *C. jejuni* cannot grow under aerobic or strictly anaerobic oxygen conditions therefore we have attempted to obtain a more complete understanding of the function and regulation of the C4-dicarboxylate transporters in *C. jejuni*.

Like in other bacteria the C4-dicarboxylate transporter genes of *C. jejuni* are dispersed over the genome (Figure 1). As they all are transcribed from different promoters, they might be regulated differently, as is seen in *E. coli*, where several transcription factors are involved in the regulation of the C4-dicarboxylate transporters in response to oxygen, C4-dicarboxylate compounds and growth phase (Janausch et al., 2002). Here we showed by real-time RT-qPCR that the oxygen concentration had only a minor but not significant effect on the transcription of the *dcuA* and *dcuB* genes in strain 81116, and that the addition of C4-dicarboxylate compounds in the complex media HI had no influence on the transcription of the C4-dicarboxylate transporter genes (Figure 2). Similar results were obtained in another *C. jejuni* strain 11168 (Woodall et al., 2005). Growth phase of the culture influenced only the transcription of the *dcuA* gene. Based on these results no transcription factor of *C. jejuni* has obtained a similar function as the *E. coli* transcription factors regulating the C4-dicarboxylates, such as FNR, ArcAB, DcuRS, or CRP (Janausch et al., 2002). Apparently, the transcription regulation in response to oxygen, C4-dicarboxylates and growth phase as seen in *E. coli* is not important for the function of the genes in *C. jejuni*.

We have shown that both the *dcuA* and *dcuB* genes are repressed and directly regulated by the RacRS system in response to low oxygen and nitrate. The *dcuA* is in most bacteria constitutively expressed and so far *C. jejuni* is the only organism in which both genes are regulated in a similar manner. The phenotypes observed for the *dcuA* and *dcuB* mutants under oxygen-limited conditions (Figure 6) were restored by the addition of nitrate to the medium (Figure 8), clearly showing that these genes are not needed under these conditions. Regulation of the C4-dicarboxylate genes by nitrate is not uncommon as the *dcuB* gene in *E. coli* is repressed by the two-component NarXL system (Overton et al., 2006). The use of nitrate as electron acceptor (E_m_ nitrate/nitrite + 430 mV) is preferred over fumarate (E_m_ fumarate/succinate + 30 mV) enabling fumarate to be used as carbon source instead of electron acceptor under oxygen-limited conditions. This explains the observed reduced secretion of succinate depicted in Figure 9.

The secretion of succinate in *C. jejuni* appeared to be mediated solely by the DcuB C4-transporter (Figure 7). In *E. coli*, the DcuB and DcuC C4-transporters, both regulated by FNR, can act synergistically to secrete succinate under anaerobic conditions (Golby et al., 1999). So far, we were unable to address the function of the two DcuC homologs in *C. jejuni* 81116 which are not co-regulated with *dcuB* and are absent in other *C. jejuni* strains (Parkhill et al., 2000; Hofreuter et al., 2006). Both DcuA and DcuB are involved in the uptake of aspartate as well as fumarate, confirming the data of Guccione et al. (2008). By regulating both DcuA and DcuB, the RacRS system completely controls the fumarate respiration in response to limited oxygen availability and the presence of nitrate.

Succinate is taken up by the DctA transporter and used as carbon source by *C. jejuni* when oxygen is not scarce (Figure 5). The DctA transporter is also needed to allow *C. jejuni* to use aspartate and fumarate as carbon source, indicating that the *dctA* mutant is unable to use or to take up these carbon sources from the medium. In *E. coli* and *Bacillus subtilis*, DctA also mediates the uptake of succinate as well as fumarate and aspartate under aerobic conditions (Davies et al., 1999; Asai et al., 2000; Janausch et al., 2002). However, no difference was observed between the *C. jejuni* wild-type and *dctA* mutant in the uptake of fumarate, suggesting that *C. jejuni* DctA is not involved in the uptake of fumarate. When fumarate or aspartate are present in the media, a large amount of energy rich succinate accumulated in the media which could not be re-used by the *dctA* mutant explaining the reduced growth of *dctA* mutant under these conditions. Beside the regulation it also appears that the function of C4-dicarboxylate genes differs in *C. jejuni*.

In our work, we highlighted the regulation and function of various C4-dicarboxylate genes in the microaerophilic bacterium *C. jejuni*. The DctA transporter is responsible for the uptake of succinate under high oxygen levels. The *dcuA* and *dcuB* genes are the only C4-dicarboxylate-regulated genes and are dependent on the two-component RacRS system in response to low O_2_ and high nitrate concentrations. DcuB is the only C4-dicarboxylate/succinate antiporter in *C. jejuni* which secretes succinate when oxygen levels are low, but is not necessary when nitrate is available.

## Author contributions

MW designed experiments, wrote the article, and performed growth experiments. JVP wrote the article, CVDL performed the High-Performance Liquid Chromatography analysis, and LVD performed all other experiments.

### Conflict of interest statement

The authors declare that the research was conducted in the absence of any commercial or financial relationships that could be construed as a potential conflict of interest.
